# Advances in Structure Modeling Methods for Cryo-Electron Microscopy Maps

**DOI:** 10.3390/molecules25010082

**Published:** 2019-12-24

**Authors:** Eman Alnabati, Daisuke Kihara

**Affiliations:** 1 Department of Computer Science, Purdue University, West Lafayette, IN 47907, USA; 2 Department of Biological Sciences, Purdue University, West Lafayette, IN 47907, USA

**Keywords:** cryo-electron microscopy, cryo-EM, density map, protein modeling, structure fitting algorithms, de novo modeling, machine learning methods

## Abstract

Cryo-electron microscopy (cryo-EM) has now become a widely used technique for structure determination of macromolecular complexes. For modeling molecular structures from density maps of different resolutions, many algorithms have been developed. These algorithms can be categorized into rigid fitting, flexible fitting, and de novo modeling methods. It is also observed that machine learning (ML) techniques have been increasingly applied following the rapid progress of the ML field. Here, we review these different categories of macromolecule structure modeling methods and discuss their advances over time.

## 1. Introduction

Cryo-electron microscopy (cryo-EM) has now become a widely used technique for determining biological macromolecular structures. Recent developments of microscopy instruments, as well as progress in image processing algorithms, have drastically improved the resolution that can be achieved by cryo-EM [1,2,3]. These advances in cryo-EM have led to the increase in the number of solved structures, particularly those which were obtained at near-atomic resolution [4,5,6,7]. According to the statistics of the Electron Microscopy Data Bank (EMDB), the public repository for electron microscopy maps, there have been 2234 maps deposited in 2019 at the time of writing, about 3.5 times the 640 maps deposited in 2015 [8]. Among these deposited maps, maps at 4 Å resolution or better increased more drastically, from 114 in 2015 to 1089 maps in 2019, which is a 9.6-fold increase.

**Figure 1:**
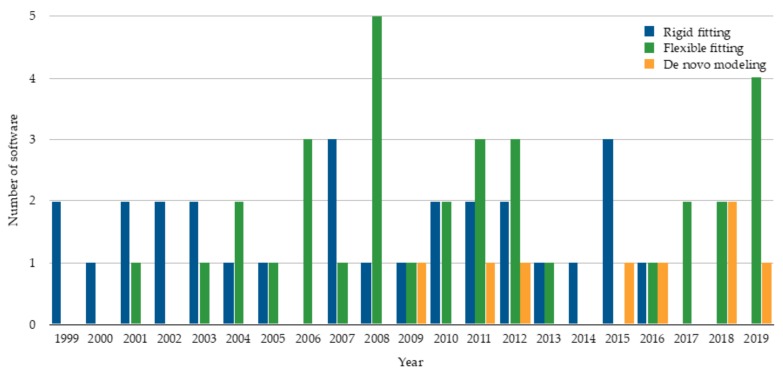
The number of rigid fitting, flexible fitting, and de novo modeling software published per year. The statistics are based on publication. The plot shows 28 rigid fitting methods [9,10,11,12,13,14,15,16,17,18,19,20,21,22,23,24,25,26,27,28,29,30,31,32,33,34,35,36], 33 flexible fitting methods [37,38,39,40,41,42,43,44,45,46,47,48,49,50,51,52,53,54,55,56,57,58,59,60,61,62,63,64,65,66,67,68,69], and 8 de novo modeling methods [70,71,72,73,74,75,76,77].

The advances in cryo-EM have certainly had a strong impact on the software developed to model molecular structures from cryo-EM maps. Structure modeling methods can be roughly classified into rigid-body fitting, flexible fitting, and de novo modeling methods. Among them, rigid fitting methods were the first to appear in literature (Figure 1). A rigid fitting method places a high-resolution structure into a low resolution EM density map. As EM map resolutions have improved, flexible fitting algorithms have emerged, which aim to consider conformational changes of rigidly fitted structures in cryo-EM maps to improve the agreement of the structures and EM maps. Recently, with an increase of need partly due to the drastic improvement of map resolution revolution, de novo modeling algorithms have started trending. De novo modeling methods benefit from the higher level of details in density, which provide information to trace protein main chain in principle without the need for known structures. Figure 1 summarizes publications of these three categories of modeling methods.

This review is intended to cover the developments of macromolecular modeling methods and the emergence of machine learning in cryo-EM analysis. The review is structured as follows: we first review the three categories of modeling methods, rigid fitting, flexible fitting, and de novo modeling methods, in this order. Then, we discuss methods that use machine learning approaches, which are emerging in recent years in the cryo-EM structure modeling field.

## 2. Rigid Fitting Methods

In rigid body fitting, high resolution atomic models which are derived from X-ray crystallography, Nuclear Magnetic Resonance (NMR) spectroscopy, or protein prediction are fitted into a cryo-EM map. One of the earliest rigid fitting methods is EMfit developed by Rossmann et al. in 2001 [12]. In EMfit, a high-resolution structure is placed manually into a specific position in the EM map. Then, a 3-D rotational search is applied to find the best orientation. After that, EMfit optimizes the initial fitting by performing local rotational and translational steps. In general, rigid-body fitting methods search for the best placement of an atomic model in a density map. Search algorithms that have been used for rigid fitting include Fast Fourier transform-based (FFT) [14,32,35], grid-threading Monte Carlo (GTMC) [16], spherical harmonic-based search [20], and geometric hashing [27]. FFT is a fast search scheme that accelerates the 3-D translational search [14]. HermiteFit speeds up the rotation step in the FFT by representing densities as three-dimensional orthogonal Hermite functions and performing rotation in the Hermite space [32]. Fast polar Fourier search is a variation of FFT, which is based on non-uniform SO(3) Fourier Transforms [35]. Its principal advantage is the ability to search efficiently and uniformly over a set of samples of the conformational space. GTMC combines grid search and Monte Carlo sampling [16]. GTMC divides the search space into grid points and uses Monte Carlo to find local maxima near the grid points to identify the global maximum. ADP_EM is a spherical harmonic-based (SH) method which applies exhaustive translational scanning and accelerates the rotational search by representing densities as SH functions [20]. Geometric hashing identifies a set of possible transformations which are stored in a fast-searchable hash map [27]. Later, the set of transformations is searched to find the best fit.

While the methods above exhaustively scan a density map for possible placements, there are methods that use other techniques to fit atomic models into density maps. One of these methods is gmfit, which converts atomic models and a 3-D density map to Gaussian mixture models (GMMs) [23,78]. Then, gmfit generates a random set of initial configurations and applies steepest-descent local searches using gradients and torques of the energy function for fitting. Pintilie et al. presented a rigid fitting protocol [25] which uses a map segmentation method, Segger [79]. Segger is based on the immersive watershed method, which sorts voxels of EM map in descending order based on their density values and assigns a voxel to a new region if it is not adjacent to another region, otherwise it is added to its adjacent region. After segmenting the EM map, Segger groups the segments using scale-space filtering [80], which moves local maxima representing regions to local density maxima in the smoothed EM maps using steepest ascent and groups regions of points converging to the same local maximum. Then, atomic models are fitted into resulted regions [25]. EMLZerD docks protein components and generates a set of different conformations. Then, docking models are ranked based on the overall shape similarity with the cryo-EM map using 3-D Zernike descriptors [30]. γ-TEMPy uses vector quantization to identify feature points in a density map, which are centers of density clusters, using a neural gas clustering technique [33]. After identifying feature points which represent the positions where atomic models can fit, γ-TEMPy applies genetic algorithm to generate different conformations.

Evaluating the quality of fitting atomic models into EM density maps can be done using different scoring functions. The scores include mutual information [33,81] and cross-correlation (CC) coefficient, which is the most widely used score. CC has been used in different forms such as density CC [25,27] which takes into account all density values, Laplacian-filtered CC [81,82], which considers only EM map contours that represent the surface of the structures, and core-weighted CC function [16] in which a core is the part where the density is least likely to be changed by other components. In addition to CC, evolutionary information such as interface conservation can be used to evaluate a set of fitted models based on the fact that interface residues are conserved higher than non-interface residue [83]. Also, surface-based scores such as normal vector score (NVA), which computes the difference in angle between the normal vectors of EM maps, and the chamfer distance score (CDAgdt), which calculates the average distance between closest surface points of two EM maps [81,82]. Another possible score is skeleton–secondary structure score that depends on matching the skeleton of detected secondary structures of the density map with the secondary structure units of the atomic model [35].

## 3. Flexible Fitting Methods

In many cases, the available conformation of component biomolecules, e.g., proteins, could be different from what the map represents for various reasons such as different functional states of a complex. Thus, flexible fitting methods are applied to change the fitted structure to conform to the EM map. Flexible fitting approaches are categorized into five major categories. These categories are normal mode analysis-based methods, molecular dynamics-based methods, geometric simulation methods, methods using structural variability of protein superfamilies, and methods guided by α-helix correspondences.

Normal mode analysis was one of the first techniques used for flexible fitting. Normal mode analysis (NMA) is a technique used to explore the natural vibrational motion of a structure [84,85]. NMA has been applied in flexible fitting in various ways. NMFF-EM is one of the NMA methods which considers only low-frequency normal modes that represent collective low-energy global motions of the biological structure. It deforms the all-atom or Cα structure iteratively along low-frequency normal modes and optimizes the overall cross-correlation between the deformed structure and the EM map [39,86]. Another NMA method, mENM, uses all normal modes, which allows it to capture both local and global structural changes, computed for a two-bead-per-residue protein representation [54]. iMODFIT uses NMA in internal coordinates (torsional space), which offers a reasonable and efficient way to search the conformational space [60].

In addition to NMA methods, molecular dynamics (MD)-based methods are well established in the field of flexible fitting. The strength of MD simulations is the use of well-established force fields, which preserves physical correctness during fitting. MDFF is an MD-based flexible fitting, which applies MD simulation that incorporates the EM density as an external potential to the molecular mechanics force field and derives the structure towards the target density [49]. There are different variations of MD-based fitting including adding biased potentials such as cross-correlation between the model and the density map [50] and symmetry information of structure [55] to enhance the fitting results. Another MD approach uses coarse-grained representation, particularly GO-model of the molecule instead of all atoms [53]. REMDFit runs MD with a number of fitting trials with different force constants to obtain sufficient conformational sampling, which are shown to be valuable especially for lower resolution EM maps [62].

Besides MD, geometric simulation is also used for flexible fitting. Geometric simulation fitting approaches change the starting structure to conform to the density map while keeping rigid bodies such as secondary structures identified early, intact during the entire simulation which maintains valid local geometry and stereochemistry [47]. Another category of flexible fitting methods uses the structural variability of protein domains of a given superfamily, according to structural databases such as CATH, to guide the fitting [42,45]. The last category of flexible fitting is α-helix correspondence-based fitting which does not require initial rigid fitting. This fitting is instead guided by the correspondence between α-helices predicted in the density map and in the model, which reduces the fitting time [63]. Overall, these different flexible fitting approaches change the conformation of a fitted structure into an EM map to improve the fit to the map. These flexible fitting methods are aimed at not only small but also at substantial domain motions, that can have a large root–mean–square deviation (RMSD) of over 15 Å.

Rigid fitting methods fit atomic structures into EM maps of intermediate to low resolution, while flexible fitting improves the quality of the fitting into intermediate resolution EM maps by performing conformational change to the atomic structure to align with the EM map structure [87,88]. The main advantage of fitting methods is that models can be built with relatively inexpensive computational cost. On the other hand, modeling is only possible when the structure is available.

## 4. De Novo Modeling Methods

Recent years have witnessed a drastic increase in the number of maps determined at a resolution of 3 to 5 Å. This is a frustrating resolution, where a part of structures can be observed in a map but difficult to build a structure model with conventional tools that are originally designed for X-ray crystallography. De novo modeling methods use maps in this resolution range, aimed at situations where known structures are not available for rigid or flexible fitting. De novo modeling tools build a full atom model or a main-chain trace without using a template structure. There are six tools that belong to this category, EM-Fold [70], Gorgon [71], Rosetta [73,89], Pathwalking [72,74], Phenix [75,77], and MAINMAST [76,90]. The methods discussed below are summarized in Table 1.

EM-Fold is designed for predicting structures of α-helical proteins from intermediate resolution EM maps [70]. First, density rods in EM map are identified manually and different secondary structure prediction methods are used to detect α-helices from the protein sequence. Then, EM-Fold places consensus predicted α-helices into the density rods using a simulated annealing Monte Carlo Metropolis search algorithm, refines the placement, and ranks the generated models. Finally, side-chains and loops are built for highest ranked models using Rosetta. Gorgon is an interactive visualization software which provides several computational tools for modeling near-atomic proteins, including tools for calculating density skeleton and matching secondary structure elements (SSE) predicted in sequence to SSE in a density map [71].

Rosetta is a software suite for modeling, predicting, and analyzing protein structures. Rosetta includes de novo modeling for cryo-EM, which consists of three main steps [73]. First, sliding a 9-residue window on the sequence and collecting representative structural fragments from databases. Second, evaluating the fragments using a 4-term score function which includes density correlation, overlap, closability, and clash terms, then finding a set of fragments that optimizes the score function using Monte Carlo with simulated annealing. These two steps are run iteratively until 70% of the sequence is covered. RosettaES overcomes the 70% covering limitation by iteratively sampling individual missing segments and combining them using a Monte Carlo assembly method [89]. Last, density-guided sampling and all-atom refinement are used to complete the partial model.

Another de novo tool, Pathwalking, is included in the EMAN package. The Pathwalking method builds a protein Cα model from an EM map using the travel salesman problem (TSP) in the following steps [74]. First, it places pseudo-atoms in the high-density regions in the density map and then applying the K-means clustering to their positions, where K is the number of amino acid residues. Next, an initial path in the EM maps is detected by a TSP solver. After that, a path refinement step is applied iteratively, identifying secondary structure elements, fixing them, and reseeding the pseudo-atoms based on that.

Phenix, a software suite for molecular structure modeling for X-ray crystallography, cryo-EM, and other methods, has a de novo modeling tool, phenix.map_to_model. Phenix.map_to_model is composed of four main steps [75]. It begins with sharpening density map to maximize its details using phenix.auto_sharpen [91]. Then, the density map is segmented to extract a unique set of connected density regions that are above an automatically determined density threshold. After that, a number of model building methods are applied to each type of macromolecule that is inferred using the phenix.guess_chain_types_from_sequences tool. Alternatively, the phenix.trace_and_build tool developed recently could be used for protein modeling [77]. Last, a model is assembled by combining structure fragments using phenix.combine_models tool, then refined using phenix.real_space_refine tool [92].

**Figure 2:**
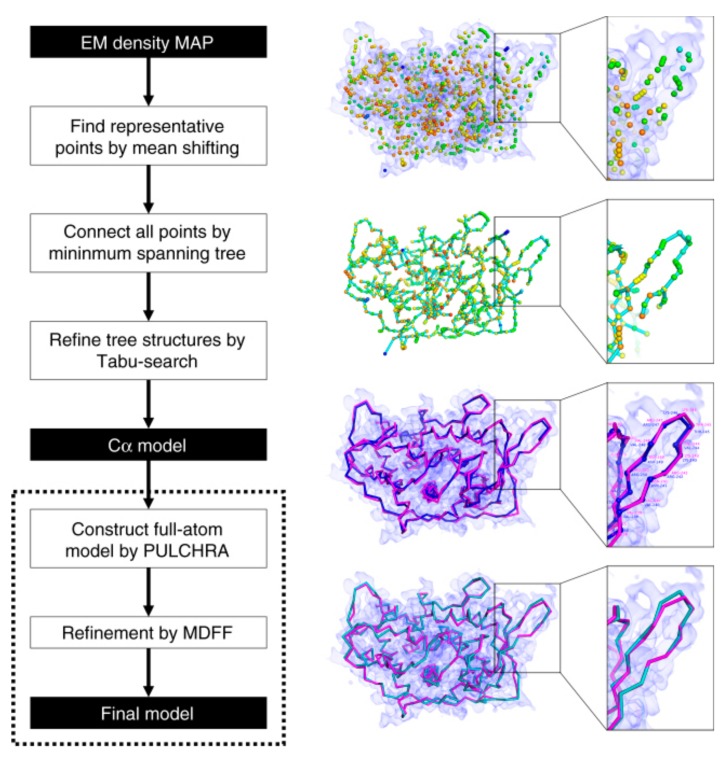
Schematic flow diagram of the MAINMAST algorithm. The cryo-EM density map shown on the right is of structural protein 5 of cytoplasmic polyhedrosis virus solved at a 2.9 Å resolution (EMD-6374). This figure was adapted from the MAINMAST paper [76].

MAINMAST (MAINchin Model trAcing from Spanning Tree) is a de novo modeling method, which was recently developed by our group [76]. MAINMAST provides a set of models with their confidence score. The procedure is fully automated and does not require any external known structures. The MAINMAST algorithm consists of six steps as shown in Figure 2. The first step is identifying local points with high density in the EM map using the mean shift method, which performs local clustering of density points. Next, identified points are connected into a minimum spanning tree (MST). Then, the MST structure is refined using a tabu search, which generates a pool of alternative trees. After that, the protein sequence is mapped onto the longest path of each MST using the Smith–Waterman dynamic programming algorithm. MSTs are ranked based on a threading (sequence-structure matching) score, which evaluates the fit of the amino acid sequence of the protein to a path in a tree. The last two steps are constructing a full-atom model for the top few hundred trees using PULCHRA [93], then refining them using MDFF [49]. The models are finally evaluated and ranked by the MDFF score. The confidence score for each local region in a model is computed as the fraction of models that have the local structure.

In general, de novo modeling methods need further development and have room for improvement. One promising strategy could be to incorporate techniques developed for protein structure prediction methods.

## 5. Machine Learning Approaches

In this section, we discuss emerging applications of ML in structure modeling for cryo-EM. ML has been actively used in many bioinformatics domains. Particularly, recently deep learning has been successfully applied to various tasks in protein sequence and structure analyses. Naturally, we have started to observe deep learning applied in software for cryo-EM, particularly in single particle picking and secondary structure prediction.

A critical step for constructing a high resolution 3-D cryo-EM map is the picking of single-particle two-dimensional (2-D) projections from thousands of 2-D micrographs. Many methods have been developed to automate the particle extraction process. ML methods applied include unsupervised clustering approaches, i.e., k-means, fuzzy c-means (FCM), and intensity-based clustering (IBC) that is used in AutoCryoPicker [94]. Recently, deep learning using convolutional neural networks (CNN) have been applied in DeepPicker [95], DeepEM [96], Deep Consensus [97], E2boxer.py procedure in the EMAN2 package [98,99], and PIXER [100].

Another task in cryo-EM where ML can be effectively applied is protein structure identification in medium to low resolution (5–10 Å) maps. At this range of the resolution, some fragments of secondary structure elements (SSE), α-helices and β-sheets are barely visible, but ML can significantly improve identification. RENNSH is a method which identifies α-helices in a density map by applying nested K-nearest neighbors (KNN) classifiers with spherical harmonic descriptors [101,102]. SSELearner, uses another classification method, support vector machines (SVM), to identify both α-helices and β-sheets in EM maps [103]. In addition to conventional machine learning techniques, very recently deep learning has been used for secondary structure prediction in EM maps. Deep learning, in particular 3-D CNN, turned out to be very suitable for identifying secondary structures from cryo-EM maps [104].

**Figure 3:**
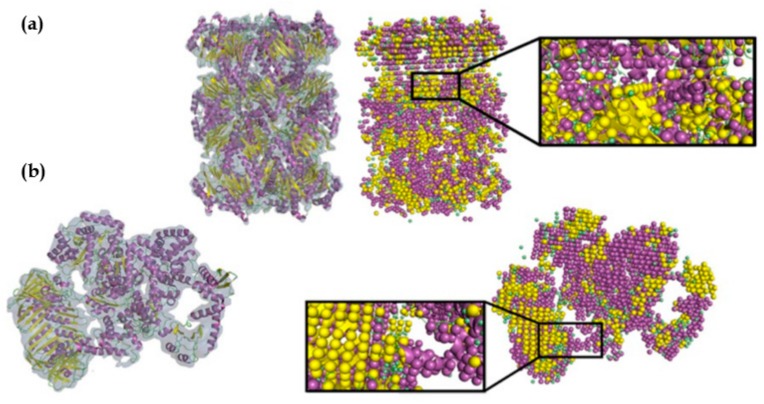
Emap2sec applied on two experimental maps. Density maps and their fitted protein structures are shown on the left and the secondary structure detection by Emap2sec is shown on the right. Spheres in magenta, yellow, and green show detected α-helices, β-strands, and other structures, respectively. (**a**) Archaeal 20S proteasome (EMD-1733 of resolution 6.8 Å; PDB 3C91). (**b**) *Eschirichia coli* replicative DNA polymerase complex (EMD-3201 of resolution 8.34 Å; PDB 5FKU). This figure was adapted from the EMap2sec paper [101].

Our group has developed Emap2sec, a deep learning-based method, which uses 3-D CNN for detecting secondary structures of a protein (α-helix, β-sheets, and other structures) in cryo-EM maps of 5 to 10 Å [101]. Emap2sec first scans a cryo-EM map with a voxel of size 11 Å. Emap2sec consists of a two-phase stacked network architecture. The first phase outputs probability values for an input voxel to have α-helix, β-sheets, and other structures, through a network with convolutional layers, a maximum-pooling layer, fully connected layers, and a softmax layer. The second phase network takes the probability values from the first phase as an input and outputs the final refined probabilities through five fully connected layers followed by a softmax layer. The purpose of the second phase is to smooth the predictions of the first phase by incorporating predictions of neighboring voxels. Emap2sec was tested on both simulated and experimental cryo-EM maps and shown to outperform existing methods. Examples of emap2sec results on two experimental maps are visualized in Figure 3. The methods discussed above are summarized in Table 2.

## 6. Conclusions

Cryo-electron microscopy is now a well-established technique for determining the structure of macromolecular complexes. At the beginning of cryo-electron microscopy, reconstructed EM images were limited to intermediate-to-low resolution, thus fitting methods of high-resolution structures into EM maps were developed. Over time, advances in cryo-electron technology enabled obtaining higher resolution EM maps, hence de novo modeling methods emerged which provided high resolution images without the need of extra resources.

New software will be required as the cryo-EM field further progresses. Two areas are expected to make substantial progress in coming years. First, as the resolution of cryo-EM improves, the study of macromolecular conformational dynamics will become possible, which requires new development of the software that enables it [87]. Macromolecules may have discrete or continuous conformational states [105]. These different conformational states, which may exist in the same sample, will need new computational approaches to be classified and extracted from a series of 3-D maps [106,107]. Second, it is highly expected that cryo-electron tomography (cryo-ET), which can analyze biological assemblies in their native cellular environment, will become a focus of studies in structural biology [108]. Recent technical advances such as focused ion beam [109] allow producing higher resolution tomograms, which would accelerate the development of cryo-ET methods.

Structural biology using cryo-EM/ET has entered an exciting era where many new experimental and computational methods are developed and synergize to produce unprecedented pictures and movies of molecules, cells, and tissues.

## Figures and Tables

**Table 1:** Strengths and limitations of de novo methods.

Methods	Strengths	Limitations
EM-Fold [70]	Able to build 3-D structure models of α-helical proteins in intermediate resolution up to 9 Å One of the pioneers in de novo methods	Models only α-helical proteins Density rods in density map are identified manually Uses external software (Rosetta) for building loops and side chains Code is not available
Gorgon [71]	Interactive software with visualization Tools for multiple steps for model structure building are provided Intended to work on maps of resolution up to 10 Å	Generates Cα-only models Human interaction is needed Due to the intended resolution, no atom level refinement provided
Rosetta [73]	Part of the Rosetta package, which has many tools for structure modeling Good local structure quality Able to handle both α-helices and β-strands Generates full-atom models	Depends on fragments retrieved from a database Has difficulty to model β-sheets Model quality deteriorates for maps at 4.5–5 Å or worse
Pathwalking [74]	Part of the EMAN2 cryo-EM modeling package Able to trace the backbone of multi-subunit complexes	Does not assign sequence to the Cα backbone models Generated models are not ranked
Phenix [75]	Part of the Phenix structure modeling package Models proteins, RNA, and DNA Generates full-atom models Tested on 476 EM maps in their paper	Model quality deteriorates for maps at 4.5–5 Å or worse
MAINMAST [76]	Generates full-atom models Does not depend on any reference structures or fragments Provides many models with a confidence score	Uses external software (MDFF, Rosetta, Phenix) to refine models Model quality deteriorates for maps at 4.5–5 Å or worse

**Table 2:** Strengths and limitations of SSE detection methods.

Methods	Strengths	Limitations
RENNSH [102]	Tested on simulated maps at 6, 8, and 10 Å, as well as experimental maps of resolutions 3.8, 6.8, and 8 Å (Algorithm: nested K-nearest neighbors classifiers)	Detects only α-helices Does not build an atomic model of predicted α-helices Limited testing on experimental maps
SSELearner [103]	Identifies both α-helices and β-strands (Algorithm: Support Vector Machines) Tested on simulated maps at 8 Å and experimental maps of resolutions (3.8–9 Å)	Does not place secondary structure elements in the density map Does not detect loops
CNN by Li. et al. [104]	Identifies both α-helices and β-strands (Algorithm: pioneer in using CNN in SSE detection)	Not tested on experimental maps Does not place SSE structures in the density map
Emap2sec [101]	Identifies three structure classes: α-helices, β-strands, and loops (Algorithm: CNN) Tested on both simulated and experimental maps of resolution up to 10 Å Code is available	Does not place SSE structures in the density map
